# Some Recent Observations in Metabolism and Their Importance in Dentistry

**Published:** 1905-06

**Authors:** Samuel A. Hopkins

**Affiliations:** Boston, Mass.


### International Dental Journal.

VOL. XXVI. JUNE, 1905. NO. 6.

### Original Communications.[1]

### SOME RECENT OBSERVATIONS IN METABOLISM AND THEIR IMPORTANCE IN DENTISTRY.[2]

BY SAMUEL A. HOPKINS, M.D., D.D.S., BOSTON, MASS.

IN order to simplify the study of metabolism it is necessary to refresh our memories with a brief reference to protoplasm and some of its properties.

The human body, as you know, is made up of various tissues which enter into the construction of the different organs of the body. We have nerve, muscular, epithelium, connective, and other tissues, each of which has its particular protoplasmic cell, differing in some slight characteristics from cells of the other tissues.

In the complicated structure of the human organism, it is difficult to study the individual cell. We have, however, in the amœba, a low form of protozoan life existing in every stagnant pool, an excellent example of a single protoplasmic cell without tissues and without organs, which has as complete a life cycle as has the complex organism which we call the human body. The amœba receives nutrition; it converts it into energy; it throws

---

[1] The editor and publishers are not responsible for the views of authors of papers published in this department, nor for any claim to novelty, or otherwise, that may be made by them. No papers will be received for this department that have appeared in any other journal published in the country.

[2] Read before The New York Institute of Stomatology, February 7, 1905.

off waste and reproduces itself, and this is life. Weismann tells us that "no amœba ever lost an ancestor by death," the parent cell merely dividing to produce two children.

The tissues of our body are made up, as I said, of these protoplasmic cells, which are receiving nutrition, converting it into energy, and getting rid of the waste.

Protoplasm is an albuminoid substance, which has no recognized histological structure of its own, yet builds up every animal and vegetable tissue. It consists of nitrogen, oxygen, carbon, and hydrogen, and a trace of sulphur in ever changeable and unstable chemical combinations.

Not to go into the recently advanced theory of electrons, it may be said that the shiftings of the molecular combinations of protoplasm produce every expression of life. The poet's dream, the artist's picture, the musician's symphony, and the knock-down blow of the prize-fighter are expressions of the ever-shifting molecular combinations of protoplasm. I have said that protoplasm was an albuminoid substance, and it is evident, therefore, that the protoplasmic cells of the body must depend upon nitrogenous food for their maintenance and activity. The proteids are the nitrogenous food substances which act as tissue-builders and furnish nutrition for the protoplasmic cells. These proteids also produce heat, especially when the carbohydrates and fats are insufficient for the purpose. They vary greatly in their solubility and in their products of decomposition.

Abdelhulden gives the following list of nitrogenous substances which have been split from the proteid molecule which they go to form: glycocol, alanin, amidovalerianic acid, leucine, phrnylalinin, glutamic acid, asparaginic acid, cystein, lysin, aginin, histidin, and others. Buchner has shown that many of these have negative chemotaxis.

Vaughan and Novy, in their excellent work on "Cellular Toxines," have pointed out the fact that many of these substances are toxines capable of great activity. This must be borne in mind, because upon these numerous products of decomposition depend some of the most serious consequences of excessive proteid metabolism. Harrington gives us examples of proteid substances: gluten flour, egg-albumin, blood-fibrin, and casein. In a general way eggs and meat have a large proportion of nitrogen, and are among our favorite forms of proteid food.

Carbohydrates may be looked upon as energy producers, and this energy, converted into heat, is measured by what we know as calories,—a calorie being the amount of heat necessary to raise one kilogram of water one degree centigrade. Fats assist in maintaining the body heat.

It is evident that before the food which we eat finds expression in the work which we do or the thoughts that we think, an infinite variety of chemical changes must take place; and metabolism may be said to be the sum of the chemical changes which take place within the body or within the cells, by which protoplasm is changed to perform special functions.

The building up of the cell we speak of as anabolism, and its breaking down as catabolism. The complete process is metabolism. The protoplasmic cell, in its absorption of nutrition, has the power of differentiation; that is to say, as the blood-stream, laden with nutrition suitable for the various needs of the body, passes through the various tissues, the muscle-cell will seize from the blood-stream that kind of nourishment which is suitable for the maintenance of muscle tissue; while the nerve-cell, ignoring the elements of muscle tissue, will seize and appropriate that which is essential for its particular needs; and so on, through all the tissues. But the presence in the blood-stream of appropriate nutritive material is not the only requirement for proper nutrition. Tired cells will not take up the nutrition presented; cells deprived of oxygen will not convert nutritive material into energy, and cells which do not receive proper exercise cannot throw off waste. And we have presented to us the necessity for normal rest, fresh air, and exercise. The inhibitive action of toxines and of diseases upon the metabolic changes in tissue-cells is very great indeed. Moreover, what I might designate as the electro-vital action of the trophic nerves, while undoubtedly dependent upon cell activity for its power, at the same time furnishes energy to the tissue-cells. You may have the arteries, veins, and capillaries of a given part in perfect condition, and filled with the best possible nutrition, and yet, if you cut off that mysterious vital energy furnished by the trophic nerves, life ceases and the part withers.

Physical chemistry, through the remarkable experiments of Professor Loeb, of California, and others, working in this field, has shown that chemistry of living matter does not differ essentially from the chemistry of inanimate objects, although the chemistry of the body is carried on at a comparatively low temperature.

It would only obstruct the easy comprehension of metabolism to dwell on the nature of the enzymes developed in the body, but it is known that these play an important part in cytology, and their action as catalyzers will doubtless explain many of the changes that go on in the tissues.

Traube discovered that every living cell behaves as if surrounded by a surface film which does not possess equal permeability for water and the substances which may be dissolved in it.

Osmotic pressure can be measured, and the study of this subject and of surface tension has led to the deduction that every particle of protoplasm which is surrounded by watery liquid has on its surface a thin film of oil. Overton has shown that herein lies the explanation of the action of alcohol, ether, chloroform, and similar substances. These have a high degree of solubility in fats, and therefore diffuse most easily in the living cells.

We know that the brain- and nerve-cells are abundantly supplied with fat, and this furnishes a possible explanation of the anæsthetic effects of the substances mentioned.

Speck has shown that when the pressure of oxygen in the air falls below one-third of its normal value, mental activity is soon impaired and consciousness is lost.

Now, before taking up the matter of food and its conversion into nutrition to meet the requirements of the human machine, I wish to express my belief, founded on a reasonably careful observation of followers of *diet systems*, that any one pursuing such a system long enough will end with chronic indigestion, just as surely as the man who follows a system in gambling will end in bankruptcy. The fruit and nut crank, the cereal crank who poultices his stomach three times a day with a mush poultice, the beef-steak and mutton glutton, are in the same danger as the quick-lunch pie fiend, or the dependent on alcoholic stimulants. A reasonable combination of all these foods might make a very appropriate dietary, but a "food hobby," except as once in a thousand times it might meet some pathological requirement, will surely overthrow its victim sooner or later.

While I am far from advocating the system of prolonged mastication introduced by Mr. Horace Fletcher, I am only too glad to pay him a fitting tribute for work which he has done in calling the attention of scientific men and the general public to the possible advantages of a smaller amount of food and a thorough mastication of that which is taken. His explanations do not bear the stamp of scientific accuracy, and his methods are extravagant and difficult to harmonize with the exigencies of our daily life, but he has compelled the attention of scientific men and led to important research work.

There is, I believe, grave danger in the Fletcher method, and instances have been lately recorded where the concentration of mind on the mastication of food and its digestion in the excessive and seemingly ridiculous manner pursued by Mr. Fletcher has led to nervous difficulties in swallowing and to severe indigestion, while the attempt to jump suddenly from a liberal diet to the minimum quantity suggested by him has led to hyperacidity of the stomach and loss of contractile power in the intestines. There is always grave danger in a too rapid change of our habits of life, but much good may come of an intelligent and reasonable attention to the almost lost art of mastication.

We, as dentists, ought to be especially grateful to any one who arouses an interest in this subject. Our respect for our work and the recognition of its importance by other people, as well as our usefulness to mankind, rests chiefly upon establishing the importance of mastication and salivary digestion.

Setting aside for the moment the subject of mastication, the interest of physiologists in Mr. Fletcher was due to the fact that here was a man in unusually good health, having extraordinary bodily energy and a clear active mind, who was subsisting on a much lower daily intake of proteid food than that which had hitherto been supposed to be essential to the maintenance of health; while at the same time, the carbohydrates, as indicated by the number of heat calories, were also considerably below the amount usually considered necessary.

Professor Chittenden, of the Sheffield Scientific School of Yale University, whose book on ‘ Physiological Economy in Nutrition” is a most important and fascinating one, had Mr. Fletcher under his observation for several months in the winter of 1902–03, and, among other things, asked him to go into the Yale gymnasium and undertake the work which the men in training for the crew were doing. To his surprise, he found that Mr. Fletcher, who was about thirty years older than any of the students, not only went through the arduous exercises of the men in training, but showed less fatigue and exhaustion than did the students.

Some of this work is embodied in an article in the *Popular Science Monthly* for June, 1903. That which appealed to the scientist was not so much the value of prolonged mastication,—which seemed especially to interest Mr. Fletcher,—but the possibility of complete and satisfactory nutrition of the body on an amount of proteid food less than one-half that which had for generations been thought essential to proper nutrition.

The standard established by Carl Voit, of Munich, has been accepted for many years. According to this standard, an adult man, weighing from seventy to seventy-five kilos (equal to about one hundred and fifty-four to one hundred and sixty-five pounds) and doing moderate work, requires one hundred and eighteen grams of proteid, or albuminous food, fifty-six grams of fat, and five hundred grams of carbohydrates, having in all a fuel value of three thousand calories.

To mark the contrast, I will say that Mr. Fletcher, at the time he was under the observation of Professor Chittenden, was taking daily an average of 44.9 grams of proteid matter, thirty-eight grams of fat, and two hundred and fifty-three grams of carbohydrates, with a fuel value of only sixteen hundred and six calories.

If, now, it could be shown that the small amount of nitrogenous food taken by Fletcher was nearer a normal standard than that established by Voit, it would mean an enormous economy in actual dollars and a great economy to the body, which is of no less importance. It is obvious that the strain on the human machine in using and disposing of excessive proteid matter must be very great indeed.

I may as well explain that carbohydrates are oxidized into carbonic acid and water, and except that these carbohydrates may cause an increase of fat which may be uncomfortable, and that they may in some cases give rise to forms of indigestion, they can do no particular harm even in excess, while the proteid foods, which are the builders of the tissues and play the most important part in cell metabolism, differ greatly, as I have said before, in their products of decomposition, many of which are undoubtedly poisonous. These products, absorbed by the intestinal wall and passing through the system in the blood-stream, are capable of seriously interfering with the functional activity of the various organs of the body, while they undoubtedly bear an important part in rendering the individual less resistant to disease.

Biliousness is a very common disturbance which may be attributed to excessive nitrogenous products. The secretions of glands are frequently altered by the poisonous products of decomposing proteids, and it has been well established that the glands which furnish the secretions for digestion are among those frequently affected. Professor Chittenden points out very clearly that the elimination of excessive amounts of these crystalline nitrogenous bodies through the kidneys places upon these organs an unnecessary burden which is liable to endanger their integrity and may result in serious injury and early impairment of function.

With these facts before him, Professor Chittenden thought it wise to ignore the suggestions made by Mr. Fletcher as to the importance of excessive mastication, because he did not wish to have too many factors to complicate his experiments. The sole object in Professor Chittenden's work was, by observations covering a long period of time and made upon different groups of men, to establish if possible a correct standard of food for the human race. It is probable, however, that the interest which had been aroused by Mr. Fletcher did lead, during these experiments, to much greater care in the mastication of food than is ordinarily used at our tables under the circumstances that usually surround us. I may say at once that in all the diets of the different groups of men experimented upon there was no attempt at restricting the kind of food. Everything imaginable entered into their bills of fare: they had coffee and sugar and fruit and cereals and starchy foods and meats, and everything that one could possibly suggest. In fact, everything was done to avoid monotony of diet. In all cases the reduction of their food was gradual. In all cases the appetite was fully satisfied. There was, of course, a tendency to reduce the quantity of meat and eggs taken, because in that way it was easier to cut down the proteids, but the diet was by no means a vegetable diet.

I can only make a brief reference to these experiments, and go on to the conclusions which seem to have a direct bearing upon our work.

The first experiment was with a group of five men who were university professors and instructors, and who took comparatively little exercise, but, on the other hand, were doing important brainwork all the time. There was great variation in age and weight. In these five cases, during the period they were under observation, there was an average excretion of less than eight grams of nitrogen, and the fuel value averaged rather less than two thousand five hundred calories, while the body weight was maintained with slight variations, as was also the nitrogen equilibrium.

You will notice that the extent of proteid metabolism is accurately determined by the nitrogen found in the urine and fæces. A proteid intake of one hundred and eighteen grams would require an excretion of sixteen grams of nitrogen. You will readily see that rather less than one-half the amount of proteid food indicated by the Voit standard was taken, while at the same time the carbohydrates were not increased. A nitrogen equilibrium briefly means that the nitrogenous food eaten should equal the amount of nitrogen excreted. If the nitrogen excreted is greater than the amount taken into the system, it would mean that the tissues were being unduly drained; but if, on the other hand, the excretion is less than the intake, it may be assumed that the system is storing up nitrogen.

These five men, during the period of from six to eight months' experimentation, expressed themselves as feeling unusually well and as having increased capacity for work, and at the end of the experiment they were unwilling to go back to their former dietary. It was impossible, as it was with the other groups of men which I will speak of later, to determine whether their power of resistance to disease was lowered by this diet, because unfortunately they all remained in perfect health. It seems, therefore, that Professor Chittenden has successfully demonstrated that a professional man may practise a physiological economy in proteid food equal to at least one-half the amount called for by the usually accepted dietary standards.

As a matter of practice, the Voit and similar dietary standards answer pretty closely to the amount which the average man consumes. From the reduction mentioned, we may reasonably expect improvement and not impairment in health, with greater capacity for work, greater resistance to fatigue, and probably to disease also.

The next group of men consisted of a detachment of thirteen from the United States Army Hospital Corps, and in their selection particular attention was paid to getting as great a variety of types as possible. In age they ranged from twenty-one to forty-three.

They weighed from fifty-two to seventy-one kilos, and they were showing at the beginning of the experiment an excretion of nitrogen equal to sixteen grams, indicating that they had been averaging an amount of proteid food at least equal to the Carl Voit standard. During the six months following they were doing daily work in the gymnasium, and besides this they had daily drill, setting-up exercises, and other light tasks; that is to say, they were in vigorous exercise. At the end of the six months' period, they were eating less than one-half the nitrogenous food called for by the usual standard, with a fuel value considerably less than the three thousand calories supposed to be necessary. These men were in better physical and mental condition in every way than at the beginning of the experiments.

So much for the soldiers. Admitting all the benefits that were derived from gymnasium work and a regular life, no one can doubt that their mental and physical improvement was due in some measure to the cutting-down of nitrogenous food.

It is also shown by these experiments that the fuel value of accepted standards is probably higher than it need be. It is, however, easier to maintain the nitrogen equilibrium if the fuel value is kept up to a reasonably high point. For the following reason I spoke in the beginning of proteids as tissue-builders and carbohydrates as energy producers, furnishing the fuel for the body. If the carbohydrates fall below the necessary requirements, the proteids will be called upon to furnish heat, and the cells will, therefore, be called upon to give up some of their nitrogen.

The next group to be experimented with consisted of eight university athletes. These were men, unlike the soldiers, in fine bodily condition, and they were exercising their brains in their studies. They were in training, and were taking an enormous amount of proteid food, since it is one of the old beliefs that athletes must be fed on a rich meat diet. Before the experiment some of these men were taking in proteids far in excess of the Voit standard. It might naturally be expected that these men would be intelligently interested in the experiments, and would give their hearty co-operation to Professor Chittenden and his work, and this proved to be the case. At the conclusion of the experiment they were excreting an average of 8.81 grams of nitrogen, corresponding to fifty-five grams of proteid metabolized, instead of double the amount which they were taking when the tests were begun. Although they all had been in training for some time before the beginning of the tests, and were supposed to be in perfect physical condition, yet every man showed improvement in his muscular power in his ability to perform his athletic feats. One man gained two championships, and another won points in track athletics for the first time, on his restricted diet.

Boils and indigestion, so common to the ordinary athlete in training, were unknown, and the capacity for mental work was well maintained.

From these experiments it is fair to deduce that a man either in athletic training or leading a professional life may with safety and, allowing for personal idiosyncrasies, with marked benefit reduce his nitrogenous food to one-half the amount which we usually take. Aside from the money-saving, which is a matter of domestic economy of great importance but not within the scope of this talk, the economy to the body in not having to care for and dispose of an unnecessary amount of food must be very great.

In nearly all the cases upon which Professor Chittenden experimented the amount of uric acid was greatly diminished. This has a direct and exceedingly important bearing upon our treatment of pyorrhœa alveolaris, and I am sorry there is not time to discuss that aspect of the question. To us, as dentists, it is enough to know that faulty metabolism leads to disease. Waste proteid matter creates injurious products of decomposition, which, taken up by the blood, are liable to produce deleterious effect upon all the tissues, including the brain and nerve-centres.

Those of us who wish to take advantage of the benefits to be derived from a reduction in the proteid food we eat, and wish to utilize this knowledge for the benefit of our patients, find ourselves deprived of the wise supervision of such a man as Professor Chittenden, and must look elsewhere for a guide to lead us to this desirable end. Such a guide we may discover in the proper mastication of all food and in reasonable common sense. It must be remembered that all changes in established habits must be made slowly, and that no departure from a generous and varied diet is at all necessary. Overconsumption of food generally comes from rapid eating. It is exceedingly difficult to eat too much if the meal be eaten slowly and each mouthful of food be thoroughly masticated. The appetite is satisfied before the danger point is reached.

It is well-nigh impossible to eat any considerable amount of very rich or highly spiced or indigestible food if you thoroughly masticate each mouthful. Try it with any rich food, and see how many mouthfuls will satisfy your appetite if they are eaten slowly, carefully masticated, and incorporated with the saliva.

(1) Thorough mastication invariably leads to the desire for simple foods.

(2) Mastication promotes the flow of saliva and accomplishes much in the digestion of starchy foods. We had grown into the habit of thinking that salivary digestion accomplished but little, and until the recent work of Dr. Cannon, of the Harvard Medical School, I think physiologists were somewhat inclined to take this view.

About the time that these experiments were being carried on by Professor Chittenden, Dr. Cannon, Assistant Professor in Physiology in the Harvard Medical School, was conducting a series of experiments which seem to throw additional light on one phase of the subject which had not been embraced in Professor Chittenden's work.

Dr. Cannon mixed with the food of cats subnitrate of bismuth, and then proceeded to study the processes of digestion with the assistance of the X-ray, the bismuth making a very satisfactory picture on the fluorescent screen. His methods are most ingenious, and his work most accurate and valuable.

We have long been accustomed to think that if starchy food escaped digestion in the mouth, there were digestive fluids that it would encounter in the intestines that would do the work of digestion. Indeed, it was felt that salivary digestion could not amount to much, because it was erroneously thought to be checked by the acid juices of the stomach. Dr. Cannon showed that while the pyloric end of the stomach had marked peristaltic movement, the cardiac end did not move in this manner. He also found that food particles taken into the stomach remained sometimes for as long a period as an hour and a half in the cardiac portion of the stomach before they became mixed with the acid digestive fluids. It was demonstrated that if food by mastication was thoroughly mixed with the saliva, the salivary digestion thus begun would continue for as long a period as one hour and a half at least after the food had been swallowed, and the vital importance of mastication of starchy foods was established.

Besides the digestion of starchy foods, (3) mastication comminutes the proteid food so that it presents a larger surface to be acted upon by the gastric juices, and relieves the stomach of unnecessary and exhausting labor. (4) It also reduces the danger of ulcers and carcinoma of the stomach to a minimum. Cannon has pointed out that ulcers of the stomach and carcinoma, which occur at the pyloric end, that end being known as the ulcer region, are caused by the rubbing over the delicate mucous membrane of that portion of the stomach of unmasticated lumps of food. They are forced down against the pyloric valve by this peristaltic motion, and then, as the intestine refuses to accept the food in such condition, are thrown back by the closing of the value, and are rubbed backward and forward until ulceration and, in some cases, carcinoma result.

At about the same time, or perhaps slightly previous to this, the medical profession, especially the surgical side of it, had begun to associate ulcers of the stomach with poor teeth, and to-day the medical profession is, by its clinical observation, ready to confirm Dr. Cannon's deduction that unmasticated portions of food produce these lesions at the pyloric end of the stomach.

I have already pointed out to this society on a previous occasion the fact that thorough mastication is the best preservative of teeth against carious action, and I will say further that I have rarely seen an instance of general and rapid carious action in the mouth of an individual who ate slowly and thoroughly masticated his food.

Whether this results from the surface polish given to the teeth or to the brushing off of the bacteria, which are destroyed by the acids of the stomach, or to improved mouth secretions as a result of the healthy stimulus given to the glands, or whether it may be due to an increase in the resistant power of the teeth themselves, the result is the same,—(5) mastication prevents decay. (6) Mastication also prevents disease. I do not speak now of the diseases of the stomach which have their origin in nervous haste or in taxing the stomach with large quantities of unprepared food, but of diseases of specific bacterial origin. We know that nearly all bacteria which produce disease find their entrance into the body by way of the mouth. I have pointed out in a previous paper that pathogenic organisms resting in unclean mouths not only multiply rapidly, but have their virulence increased, and I referred to the pneumococcus as one especially influenced by environment. It seems a far cry from mastication to pneumonia, but it is probably true that the destruction of bacteria by the acid secretions of the stomach goes far to limit disease. This was ably demonstrated by Koch many years ago in the study of the cholera bacillus, and it has been since shown that prolonged mastication, particularly of hard substances, goes far to free the mouth of bacteria. Bacteria are most numerous in the mouth before eating and fewest just after a meal, and the thorough cleansing of the mouth by vigorous mastication must assist in the removal of pathogenic bacteria.

Added to this is the fact that bacteria do not pass through healthy epithelial tissue, but irritated, inflamed, or diseased epithelial tissue lends itself readily to the passage of bacteria, and this latter condition of the alimentary tract may easily be brought about by improper feeding.

(7) The exercise of mastication, besides producing healthy glandular secretions, insures an active and healthy circulation of blood through the tissues of mouth and adjacent parts. It promotes the flow of lymph and secures the healthy nutrition of the teeth themselves.

If this be so in adult life, what must it mean in childhood? The development of the bones of the face and of the teeth, the development of the muscles, and the health of the soft tissues must be very greatly affected by the influence which exercise or its lack affords. There are many serious students of this subject who believe that contracted jaws and accompanying irregularities, adenoid growths, and other diseases of the soft tissues, as well as weakness of the teeth themselves, are largely due to the lack of mastication which our present method of feeding children involves.

I think I have said enough to demonstrate the importance of mastication, but it is interesting to see how, from different points of view, the same conclusions may be arrived at.

In the first place was Mr. Fletcher, who insisted on excessive mastication, and thought that because of excessive mastication smaller amounts of food were needed; then Professor Chittenden, who has demonstrated, without particular attention being given to mastication, that smaller amounts of food are required than we had supposed; then Dr. Cannon's excellent work, showing the danger, from another point of view, of swallowing unmasticated food and the possibilities of salivary digestion if the food was thoroughly masticated; and then the clinical evidence provided by the medical profession of the results of bolting our food.

I should like to include in this paper something of Professor Pawlow's work on nervous and psychological influences of digestion, but I have already taken too much of your time.

In conclusion, I think it will be admitted that in the conservation of the organs of mastication we as dentists have a life work second in importance to no other, and as these truths which I have brought to your attention become more and more recognized, the respect and admiration of the world for our profession will be greater than ever before. It is by the conscientious study of these problems that we make ourselves worthy of the respect and confidence which the public reposes in us.

